# Evaluation of dose point kernel rescaling methods for nanoscale dose estimation around gold nanoparticles using Geant4 Monte Carlo simulations

**DOI:** 10.1038/s41598-019-40166-9

**Published:** 2019-03-05

**Authors:** Sandun Jayarathna, Nivedh Manohar, Md Foiez Ahmed, Sunil Krishnan, Sang Hyun Cho

**Affiliations:** 1 Department of Radiation Physics, The University of Texas MD Anderson Cancer Center, Houston, Texas USA; 2 Department of Radiation Oncology, Emory University, Winship Cancer Institute, Atlanta, Georgia 30322 USA; 3 Department of Radiation Oncology, The University of Texas MD Anderson Cancer Center, Houston, Texas USA; 4 Department of Imaging Physics, The University of Texas MD Anderson Cancer Center, Houston, Texas USA

## Abstract

The absence of proper nanoscale experimental techniques to investigate the dose-enhancing properties of gold nanoparticles (GNPs) interacting with radiation has prompted the development of various Monte Carlo (MC)-based nanodosimetry techniques that generally require considerable computational knowledge, time and specific tools/platforms. Thus, this study investigated a hybrid computational framework, based on the electron dose point kernel (DPK) method, by combining Geant4 MC simulations with an analytical approach. This hybrid framework was applied to estimate the dose distributions around GNPs due to the secondary electrons emitted from GNPs irradiated by various photon sources. Specifically, the equivalent path length approximation was used to rescale the homogeneous DPKs for heterogeneous GNPs embedded in water/tissue. Compared with Geant4 simulations, the hybrid framework halved calculation time while utilizing fewer computer resources, and also resulted in mean discrepancies less than 20 and 5% for Yb-169 and 6 MV photon irradiation, respectively. Its appropriateness and computational efficiency in handling more complex cases were also demonstrated using an example derived from a transmission electron microscopy image of a cancer cell containing internalized GNPs. Overall, the currently proposed hybrid computational framework can be a practical alternative to full-fledged MC simulations, benefiting a wide range of GNP- and radiation-related applications.

## Introduction

Gold nanoparticles (GNPs) have emerged as promising sensitizers of tumors to radiation therapy. The effectiveness of GNPs for this purpose has been demonstrated well in preclinical animal studies reporting considerable delay in tumor regrowth and a remarkable increase in the survival of animals after the injection of GNPs^1–5^. The primary physical mechanism of GNP-mediated radiosensitization observed during photon irradiation is thought to be an increase in the secondary electron production due to the larger photoelectric cross-sections of gold compared with tissue or water^6,7^, leading to considerable (>100%) local radiation dose enhancement around GNPs^8,9^. Depending on the energy of incident photons and the medium of interaction, these secondary photo- or Auger-electrons can cause DNA damage (either directly, by hitting DNA strands, leading to breakage; or indirectly, by producing free radicals that can break DNA), which ultimately results in cell death^10–14^. Therefore, quantifying the nanoscopic dose distribution (or energy deposition) around a given GNP geometry or distribution during photon irradiation is an essential step to predicting biological outcomes due to GNP-mediated radiosensitization^4,8,15–17^.

Due to the current difficulty in accurately measuring dose distributions on the nanoscale, researchers have attempted to quantify the dose distributions around different GNP configurations (e.g., single, clustered) using various computational techniques, such as Monte Carlo (MC) and analytic methods^8,9,18–22^. The results obtained from MC methods are generally considered to be accurate within the statistical and inherent (e.g., interaction cross-sections) uncertainties, although nanoscale MC simulations require a well-defined three-dimensional (3D) geometry as well as extensive computational resources and time. On the other hand, analytical methods allow relatively fast calculations as well as the use of a two-dimensional (2D) geometry which can be further reduced to one-dimension (1D) (e.g., when radial symmetry exists). Despite these advantages, they provide less accurate results (depending on the complexity of the problems) than MC simulations, due to their inability to fully take into account detailed physical effects such as particle interactions, energy depositions, secondary particle productions, and reflection of particles at the material boundaries. Thus, the decision of whether to use an MC or an analytical method needs to be made, considering the trade-off between available computational resources and the required accuracy for the problem in question. For example, it is not only challenging but also computationally expensive to perform a complete MC simulation to calculate the intracellular dose distribution due to photon irradiation using a realistic cellular geometry model that includes internalized GNPs. Therefore, it is important to investigate nanoscale dose calculations based on analytical methods, which can be acceptable under many practical situations, as reasonable alternatives to MC-based calculations.

The main objective of the present study was to develop a hybrid computational framework by combining MC and analytical methods, which can be used to approximate the dose distribution around a single GNP or a cluster of GNPs, rather than calculating the dose distribution for every GNP geometry and distribution. The MC component of this framework was based on the Geant4 (G4) particle transport toolkit^23^, and analytical calculations were based on well-established electron dose point kernel (DPK) methods^24–27^. The applicability of the electron DPK method is governed by the associated assumptions used to derive the kernel, mainly that the medium used to extract the absorbed dose around a point isotropic electron source should be infinite and homogeneous^28^. For example, in clinical dosimetry calculations, electron or beta DPKs are used by assuming the region of interest is a homogeneous entity and neglecting any tissue or bone density variations^29^. In situations in which homogeneity within the region of interest is perturbed by large density fluctuations, the DPK method fails to extend its capability of estimating the dose beyond the boundary at which the homogeneity is perturbed. This inability to properly handle the dose perturbations in heterogeneous situations remains the main limitation of the DPK method in dosimetry applications.

To our knowledge, analytical methods that can extend the accuracy of the DPK method in the above-mentioned heterogeneous situations have yet to be developed. However, a few researchers have investigated the applicability of the DPK method in such situations using semi-empirical methods that utilize some rescaling techniques^28,30,31^. These rescaling techniques exploit the physical properties of the materials involved and modify the path lengths traversed by electrons in different media based on the equivalent pathlength approximation (EPA). The most common rescaling factors used with the EPA-based methods are the linear range ratio (LRR) of water to the medium^32^ and the physical density ratio (PDR) of the medium to water^33^. In the LRR method, the ratio of the continuous-slowing-down approximation (CSDA) electron range between a material of interest and water at a certain energy is used to rescale the path length traversed through the medium. In effect, the distance the electrons propagate through a material is equivalent to the distance in water multiplied by the range ratio between the two media. The limitations of these rescaling methods for millimeter-sized spherical geometries were first investigated using MC simulations for different materials, showing that the discrepancy between MC simulations and DPK rescaling methods increases with the atomic number of the spherical object (up to 100% for extreme cases)^28^.

In the present study, we assessed the applicability of the aforementioned DPK rescaling methods for nanoscale dosimetry calculations dealing with different GNP configurations embedded in a homogeneous water/tissue medium. Additionally, we investigated a novel DPK rescaling method based on the total stopping power (TSP). The CSDA, because it does not take into account energy fluctuations, is an approximation of the range of an electron; thus, it is important to examine other possible rescaling factors that depend more closely on the electron interactions causing energy loss. When an electron interacts with a medium, it loses energy because of inelastic (e.g., collisional and radiative) interactions. This is given as the TSP of the electron for a given medium at a particular energy. Based on these considerations, we introduced TSP in this study as an alternative energy-correlated rescaling factor.

Overall, we considered two different types of EPA-based approaches: i) geometry- and ii) material-based rescaling coupled to a particular rescaling factor. The accuracies of all DPK rescaling methods, along with different factors, were compared with full-fledged G4 MC simulations. Finally, the appropriateness of the hybrid method over full-fledged G4 simulation was examined for clustered GNPs present inside a cancer cell using a transmission electron microscopy (TEM) image that was properly demarcated into gold and water voxels.

## Results

### Derivation of the homogenous DPK

**Figure 1:**
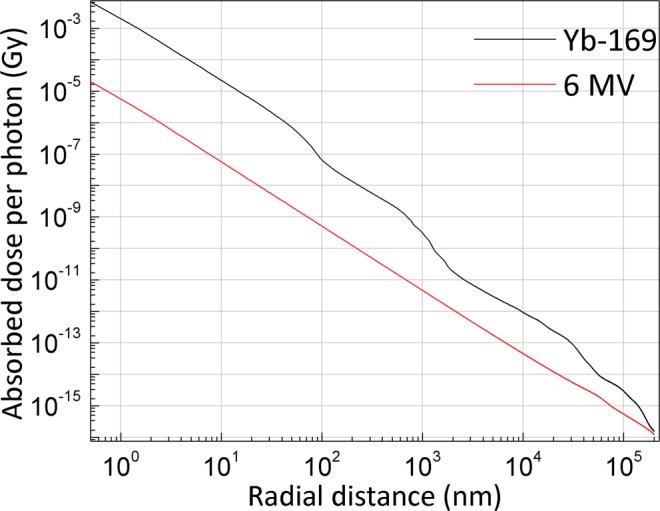
Electron DPKs as a function of radial distance for Yb-169 and 6 MV sources. The results were normalized to the number of photons simulated. For the clarity of visualization, the curves shown in this figure were terminated at 200 µm.

The homogeneous electron DPKs in water for the two different photon spectra normalized to the number of incident photons are shown in Fig. 1. A sharp decline in the DPK was noticed for Yb-169 compared to 6 MV. The extent of the DPK, which depends on the effective range of the secondary electrons produced, was up to 200 µm for Yb-169 and up to a few millimeters for 6 MV. The magnitude of the DPKs clearly differed. As shown, the secondary electrons due to photon irradiation using a Yb-169 source (“Yb-169 irradiation”) deposited more dose per incident photon than those due to irradiation using a 6 MV photon beam (“6 MV irradiation”) did over the given distance range. These observations agreed very well with previously published results^8^.

### Applicability of DPK for non-planar boundary

**Figure 2:**
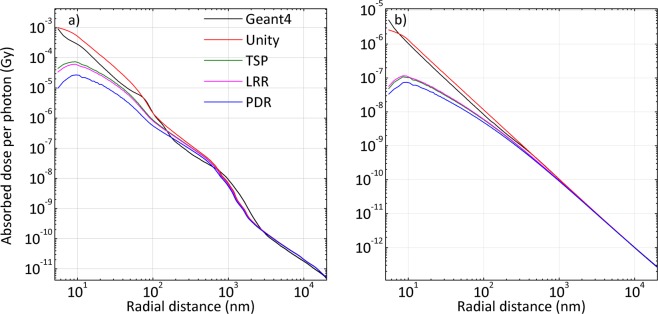
Comparison of the absorbed dose distribution around a GNS with a 5-nm radius (non-planar boundary) by MC and DPK/rescaling methods during (**a**) Yb-169 irradiation and (**b**) 6 MV irradiation.

For a gold nanosphere (GNS) with a radius of 5 nm, the results based on the aforementioned rescaling factors and G4 simulations for Yb-169 and 6 MV are compared in Fig. 2(a,b), respectively. The profiles based on rescaling of the DPKs agreed reasonably well with the G4 simulation results and almost overlap with them after 1000 nm or 1 µm. The results for all geometries for both photon sources are shown only to 20 µm from the surface, which is the approximate diameter of a HeLa cell. All other considered GNS geometries (r = 10, 15, 25, 50, 100 and 250 nm) for Yb-169 and 6 MV are shown in Supplementary Figs S1 and S2, respectively.

Some qualitative observations can be made from the above results. One of the clear tendencies in the results is that, in all considered geometries, the agreement between the G4 simulations and rescaling methods based on TSP and LRR was always better than that of the results derived from the PDR rescaling factor for Yb-169. However, the results based on the unity rescaling factor (i.e. geometry-based rescaling only) showed the best agreement among all the rescaling factors for 6 MV at all GNS radii, while the disagreement for Yb-169 based on the same method was significantly higher for GNSs with larger radii. For example, during 6 MV irradiation in the immediate vicinity of the GNS boundary (<100 nm), the maximum discrepancies between G4 simulation results and unity rescaling factor for radii of 5 nm and 250 nm were ~35% and ~70%, respectively, whereas during Yb-169 irradiation, they were ~80% for a radius of 5 nm but 200% for a radius of 250 nm. On the other hand, during the Yb-169 irradiation of GNSs with larger radii, the discrepancies based on the TSP and LRR rescaling factors suggested an acceptable agreement (a maximum discrepancy from the immediate vicinity of ~70–75% for TSP and LRR), even for radii of 100 nm and 250 nm (Supplementary Fig. S2(e,f)). The computation time for G4 simulations was around 30 hours for Yb-169 irradiation and marginally higher (40 hours) for 6 MV irradiation on a single node of a high performance computing (HPC) cluster with 24 processing units. In contrast, the DPK rescaling method in conjunction with all the rescaling factors needed just 15 hours for the same cases on a single node with 10 processing units (Supplementary Fig. S3).

### Applicability of DPK for planar boundary

**Figure 3:**
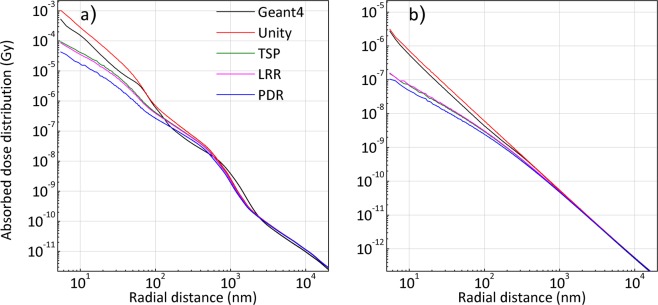
Comparison of the absorbed dose distribution for the planar boundary (long-axis) of a GNR with a 1:1 diameter-to-height ratio (10 nm × 10 nm) by MC and DPK/rescaling methods during (**a**) Yb-169 irradiation and (**b**) 6 MV irradiation.

The results along the long-axis of gold nanorod (GNR) with an aspect ratio of 1:1 (diameter-to-height ratio of 10:10 nm) involving a planar boundary (i.e., top and bottom of the nanorod) for Yb-169 and 6 MV are shown in Fig. 3(a,b), respectively. The results for the remaining GNR aspect ratios (1:2, 1:3 and 1:4), which reflect the diameter-to-height ratios of 10:20, 10:30, and 10:40 nm for Yb-169 and 6 MV irradiations are given in Supplementary Fig. S4.

For Yb-169 irradiation of all considered aspect ratios of GNRs, the results derived from the unity rescaling factor generally agreed better with the G4 results at short radial distances (over 5–20 nm and 40–100 nm) from the surface of GNR, compared with the results derived from all other rescaling factors. On the other hand, the results derived from the unity rescaling factor over a specific range (20–40 nm) of radial distances deviated more (maximum discrepancy ~80%), compared with the results calculated by TSP and LRR (maximum discrepancies ~50–55%). Over the radial distance range of 100 nm–1 µm, the results based on TSP and LRR always agreed better, compared with the results from unity scaling factor.

For 6 MV irradiation, the results derived from the unity rescaling factor showed a better agreement (maximum discrepancy of 40%) compared with the calculations of TSP and LRR. Among the rescaling methods applied for Yb-169 and 6 MV irradiation, the results derived from PDR showed the worst agreement with the G4 results for all geometrical configurations.

### Comparison of the different rescaling methods

**Figure 4:**
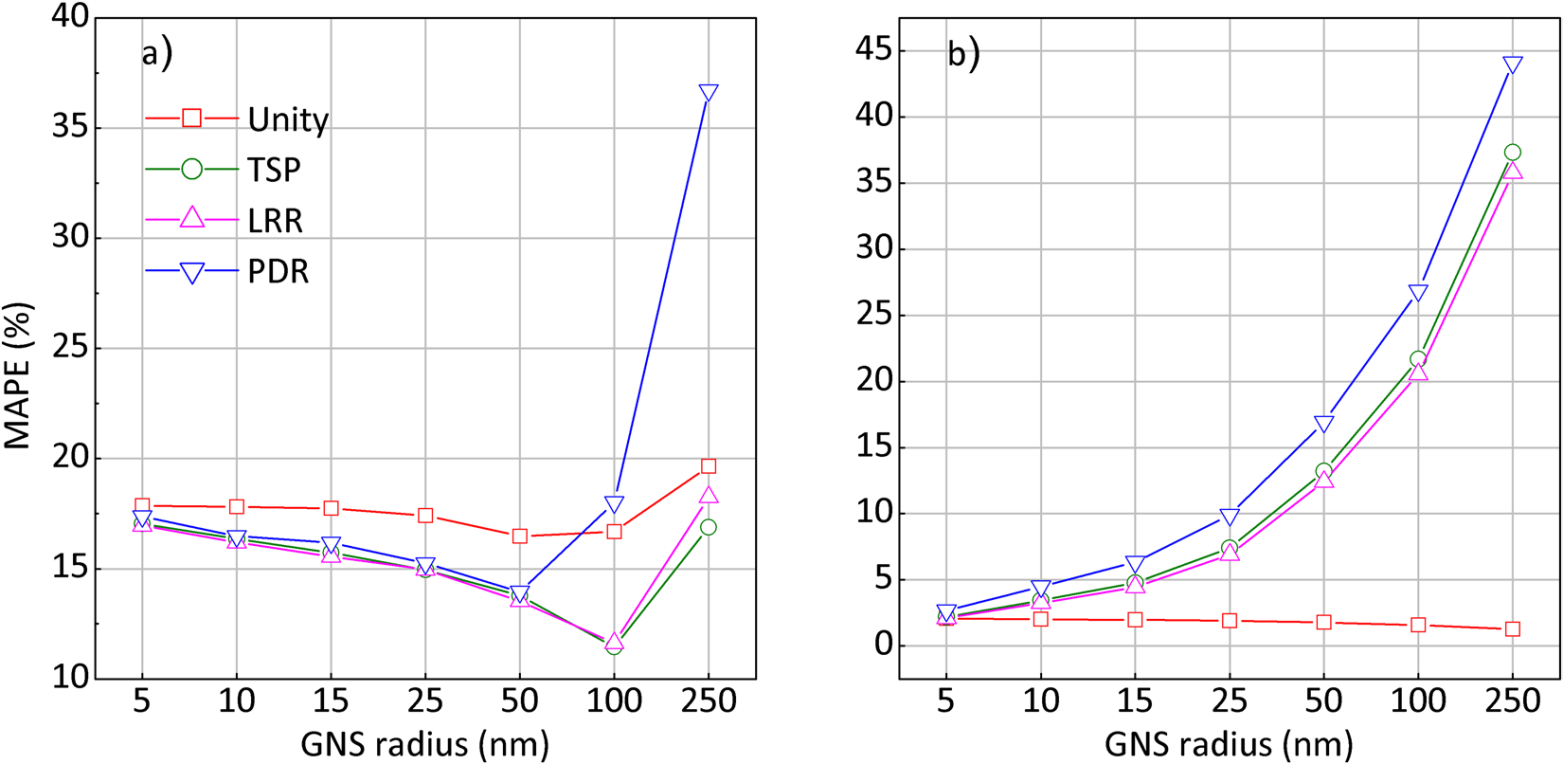
MAPE curves for all GNS geometries during (**a**) Yb-169 and (**b**) 6 MV photon irradiation.

For GNS geometries, the calculated mean absolute percentage error (MAPE)^34^ values for Yb-169 and 6 MV results based on all the rescaling factors are summarized in Fig. 4(a,b), respectively. For Yb-169 irradiation, the TSP/LRR-based rescaling method resulted in the minimum discrepancy of 11% for 100 nm-radius GNSs. Even for very large GNSs (r = 250 nm), the results based on TSP or LRR were found to match the G4 simulation results with less than 18% of discrepancy. Overall, the DPK results based on the unity rescaling factor showed larger discrepancy around 17–20% for all considered GNS geometries. This suggests that applying geometry-based rescaling only fails to properly reproduce the G4 results for Yb-169 irradiations when larger GNSs are considered.

For 6 MV irradiation, on the other hand, the mean deviations resulting from the unity rescaling factor were found to be less than 5%, showing a remarkable agreement with the G4 simulation results for all considered geometries. For all other rescaling factors, the discrepancies increased with increasing GNS radii. Among the rescaling methods considered, PDR showed the highest discrepancy up to 45% for all geometries, while TSP and LRR showed discrepancies that were smaller than those for the PDR. These results suggest the necessity of energy-dependent rescaling methods for Yb-169 irradiation, whereas only a geometry-based rescaling appears to be sufficient to reproduce G4 simulation results for 6 MV photon irradiation.

**Figure 5:**
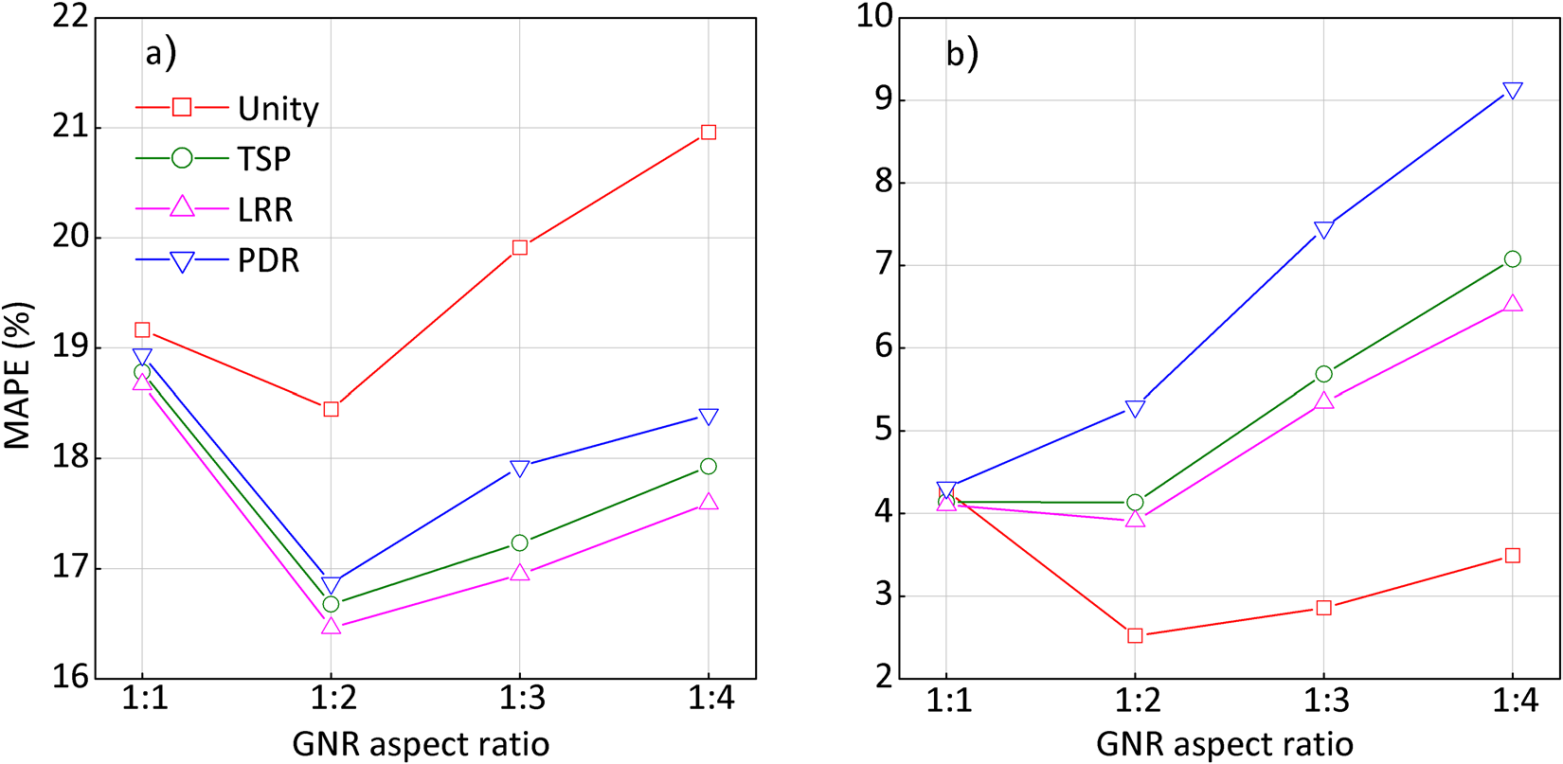
MAPE curves along the long axis of all GNR geometries during (**a**) Yb-169 and (**b**) 6 MV photon irradiation.

The evaluated MAPE values for the planar boundaries during Yb-169 and 6 MV irradiation are shown in Fig. 5(a,b), respectively. During Yb-169 irradiation, the discrepancies resulting from the unity rescaling factor for all considered geometries were 19–21%. For the TSP and LRR rescaling factors, discrepancies of 17–19% were noted. The deviations for 6 MV irradiation were relatively lower than those for Yb-169 irradiation. Specifically, the unity rescaling factor for all geometries resulted in a discrepancy of less than 4%, which was similar to the level found for GNS, whereas other rescaling factors led to larger discrepancies of 4–9%. In all considered geometries and photon sources, the rescaling based on PDR was found to be the least similar to the G4 simulation results.

### Simulation of clustered GNPs present within a cell

**Figure 6:**
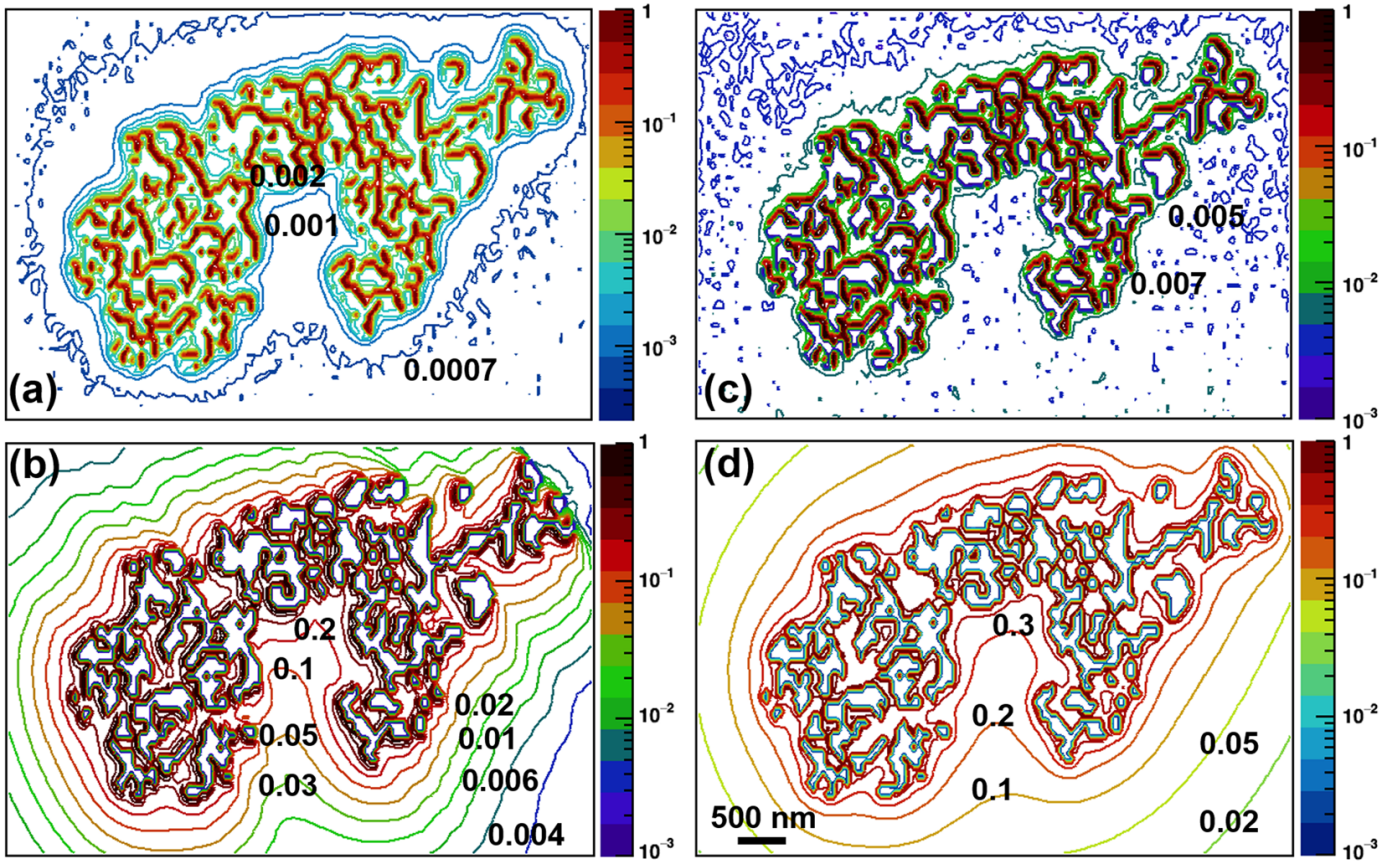
Relative dose distributions for Yb-169 and 6 MV photon irradiation using G4 simulations and DPK/rescaling methods. (**a**) G4 simulation for Yb-169. (**b**) DPK method with LRR rescaling factor for Yb-169. (**c**) G4 simulation for 6 MV. (**d**) DPK method with unity rescaling factor for 6 MV.

The dose distribution around intracellularly present GNP clusters irradiated by both photon spectra was calculated from the G4 simulation and DPK/rescaling method, as shown in Fig. 6(a–d). The dose at each pixel was normalized to the maximum dose to yield the relative dose distribution, which is represented by the color bar. Differences of one or two orders of magnitude were found between the DPK-based approach and the G4 simulation results. For example, in the immediate vicinity of the GNP cluster for Yb-169, G4 simulation yielded a relative dose of ~0.001–0.002, while LRR-based rescaling resulted in a relative dose of ~0.1–0.2. However, we note the differences shown here were not entirely due to the discrepancies between the DPK-based approach itself and G4 simulations because of the different irradiation conditions involved in the current examples (e.g., internal electron vs. external photon sources, point isotropic emission vs. directional emission of the secondary electrons). Thus, qualitative comparisons between the results would be of more merit within the current scope. Qualitatively, both G4 simulations and rescaling methods showed appreciable dose deposition in the immediate surroundings of GNPs. Also, the shape of the iso-dose curves in the immediate vicinity of the GNP cluster obtained from both DPK/rescaling methods was comparable to that from G4 MC simulations. Besides, the DPK/rescaling methods produced well-defined iso-dose curves across the considered region of interest around the GNP cluster, whereas G4 MC simulations did not.

## Discussion

### Extension of the DPK rescaling methods for heterogeneous media

Several assumptions were made during the formulation of the rescaled DPK method. Of note, it was assumed that electron transport in gold could be approximated by adequate scaling of electron transport in water. Within the scope of the current work, the accuracy of this assumption depends on the degree of electron scattering occurring inside, outside, and at the boundary of the GNP, causing energy loss due to ionization and *bremsstrahlung* photon production. Another assumption used to derive the rescaled DPK was that electron backscattering was absent from the surface of the GNP. This backscattering may differ depending on the specific shape of the boundary. For example, it may be greater for a planar boundary than for a non-planar boundary, and DPK-based rescaling methods must properly account for this. Therefore, these assumptions introduce errors, which were observed for all GNP geometries considered in this study.

One possible situation that minimizes the discrepancies due to the aforementioned assumptions is in the case involving the least possible perturbation, which is the smallest-scale geometry considered in this study. Within the smallest-scale geometry, the electron scattering, backscattering, and energy loss can be assumed to be minimal, and the least deviations with respect to G4 simulations can be expected. For Yb-169 irradiation, this is evident for GNSs with 100 nm or smaller radii (MAPE around 11–17%).

In particular, 6 MV photon irradiation involves low scattering cross sections and relatively straight particle track structures of secondary electrons, compared with Yb-169 irradiation. This is the main reason the results based on the unity rescaling factor (no rescaling or geometry-based rescaling only) were found to predict 6 MV results more accurately than other rescaling factors for all considered geometries in this study. For all geometries and incident energies considered in this study, the TSP and LRR rescaling factors were found to be more reliable than the PDR rescaling factor. The main reason for this better agreement is that both TSP and LRR used the secondary electron energy coupled with radiative energy loss and collision energy loss, which have more energy dependence than does using just the ratio of the physical densities of the media.

In the case of GNRs, the proposed method to calculate the dose distribution around different axis-systems was found to be useful for further understanding of the dose distribution around cylindrical geometries. Well-separated discrepancies of the predicted dose profiles from rescaling factors were found for different source-target geometries, in which the planar boundary has a relatively small discrepancy with respect to the non-planar boundary in both forms of photon irradiation. This disparity, found in different curvatures, may be due to the way G4 accounted for the emission of electrons in curved surfaces and flat surfaces from inside the GNP region to the water region. The discrepancy in GNRs increases as the aspect ratio increases, due to the increasing length of an electron’s path to the scattering medium region along the major-axis region.

### Simulation of clustered GNPs present within a cell

In principle, the DPK method described in this study can be extended to any geometry, including irregularly shaped GNP clusters, which are hallmarks of GNPs internalized by cells but have not yet been adequately addressed in GNP-related computational studies. Our objectives in comparing G4 simulations and the DPK/rescaling method for an actual GNP cluster were to estimate any limitations of both methods and assess the efficiency of the EPA-based techniques to handle complex nanoscale geometry. As illustrated in the Methods section (see “Dosimetry calculations with clustered GNPs within a cell”), the G4 simulation provides the more realistic particle track structures and interactions. For example, most secondary electron tracks produced inside a GNP get absorbed before leaving the GNP region, whereas all the tracks (rays) originating in DPKs contribute to the dose calculation in each water pixel outside the GNP regardless of the electron scattering or absorption occurring within the GNP. This is the main reason for the statistically poor (low) counts in the full-fledged G4 simulation results compared with the well-defined iso-dose distribution resulting from the DPK/rescaling method.

One of the main advantages of using the DPK/rescaling method compared to a full-fledged MC simulation for complex GNP cluster geometry is the fast accumulation of dose values in water voxels near and away from the GNP region. For example, with 60 billion photons introduced onto the GNP geometry, which ran for about 3 days on a dedicated HPC cluster, the G4 MC simulation was barely able to score dose depositions in half of the available water voxels, as shown by the fewer contour lines away from the GNP cluster in Fig. 6. (a), (c) for Yb-169 and 6 MV, respectively. On the other hand, the DPK/rescaling method was able to calculate the dose deposition in each water voxel around the GNP geometry on a single core processor within three hours for LRR-based rescaling and approximately 30 minutes for geometry-based rescaling. Another convenience of the DPK/rescaling method over G4 MC simulation is that minimal pre-processing of geometries is required for dose calculations. Specifically, preparation of simulation components and post-processing of G4 simulation require several additional steps, making the entirely G4 simulation–based approach computationally more intensive than the DPK/rescaling-based approach. Overall, the DPK/rescaling method was found to be more straightforward and computationally efficient to handle irregularly shaped internalized GNP clusters for the nanoscale dose calculations, albeit the difficulty in estimating its accuracy.

Unlike the 6 MV case in which one can use the unity rescaling factor for the gold medium during the computation, the Yb-169 case requires a proper rescaling of the DPK using TSP or LRR. Applying such rescaling at keV energies will require additional computation time and a pixel-by-pixel comparison to find the gold medium along the trajectory. This was the reason for marginally higher computation time for LRR-based rescaling method in Yb-169 over no rescaling in 6 MV. Future work must investigate the currently shown disparity further. Alternative solutions might include using the ratio of energy deposition inside and outside GNPs as a function of the GNP size to rescale the DPKs or considering the decrease in electron energies as they traverse matter. However, these solutions would necessitate more convoluted approaches to model and validate.

In this study, we only considered photon irradiation along the positive y direction, and the scattering, absorption, and emission of secondary electrons may be different for the same photon beam depending on the incident beam orientation. Further studies may be needed to fully understand the limitations of the full G4 simulations in this type of two-dimensional complex GNP cluster situation. On the other hand, no such directional dependency is needed in the DPK/rescaling method. Thus, the results presented herein should be valid only for the considered clustered geometry, and observations may be different for a geometry derived from a different TEM image. For example, both methods may require additional computational resources, time, and pre-processing for a very complex geometry with more GNP pixels.

Overall, while they have some limitations as discussed above, the currently investigated approaches are readily available and can be useful for many research tasks involving GNPs and radiation. For example, they can facilitate the design and fabrication of GNPs for radiation-related applications (nuclear medicine, radiotherapy, etc.) as well as modelling of GNP-mediated radiosensitization, especially when full-fledged track structure MC simulations are not possible (e.g., only 2D geometry is known) or computationally too expensive to perform (for realistic clinical applications).

## Methods

### Dose point kernel rescaling method

The proposed computational technique was implemented in three independent steps. In the first step, the electron DPK was acquired in a homogeneous water medium by G4 MC simulations. In the second step, the homogeneous medium was perturbed to a heterogeneous source-target geometry by introducing a GNP at the center of the water medium; for this instance, the initial DPK in water is no longer applicable. In the third step, a rescaling method coupled to a particular rescaling factor was used to integrate the initial DPK to derive the radial dose profile beyond the boundary of the GNP. Since it was impractical to validate the rescaling methods for all possible geometrical shapes and curvatures available, we used the non-planar curvature present in gold GNSs and the planar curvature along the main axis of GNRs, both of which are widely used geometries for GNP-mediated radiosensitization studies.

We investigated the applicability of four different factors for rescaling of the homogeneous/water DPK. The first factor is independent of the heterogeneity and electron interactions (i.e., no rescaling) and taken as unity. The second and third factors are based on the correlation of heterogeneity with electron interactions through LRR and TSP ratio, respectively. Finally, the fourth factor depends on only the heterogeneity of the medium and is derived from the PDR.

### Monte Carlo simulations

#### Physics selection and particle transportation

We used the G4 toolkit because of its full customizability with object-oriented programming, free availability, and in-built multi-processing architecture, which is fully compatible with modern HPC clusters. Any G4 simulation must have a geometry description (GNP and water medium) and a physics model to explain the particle-medium interactions (photon and electron interactions with gold and water). In the current investigation, we used the G4 low-energy Penelope physics model. The selection of the Penelope model was based on previous findings of its good agreement with other MC models in nanodosimetric calculations^35^ as well as DPK calculations in water^36^. Photoelectric effect, Compton scattering, and pair production were used as the default physical interaction processes during photon transport. The Auger electron emission was activated as explained elsewhere^37^ during the photoelectric effect. The energy production threshold for secondary electrons was set to 250 eV for both gold and water media (~1.8 nm and ~3.7 nm range in gold and water respectively). Electrons were transported in water and within the GNPs by activating multiple Coulomb scattering, electron ionization, and bremsstrahlung photon production with an energy threshold of 250 eV.

#### Simulation geometry

**Figure 7:**
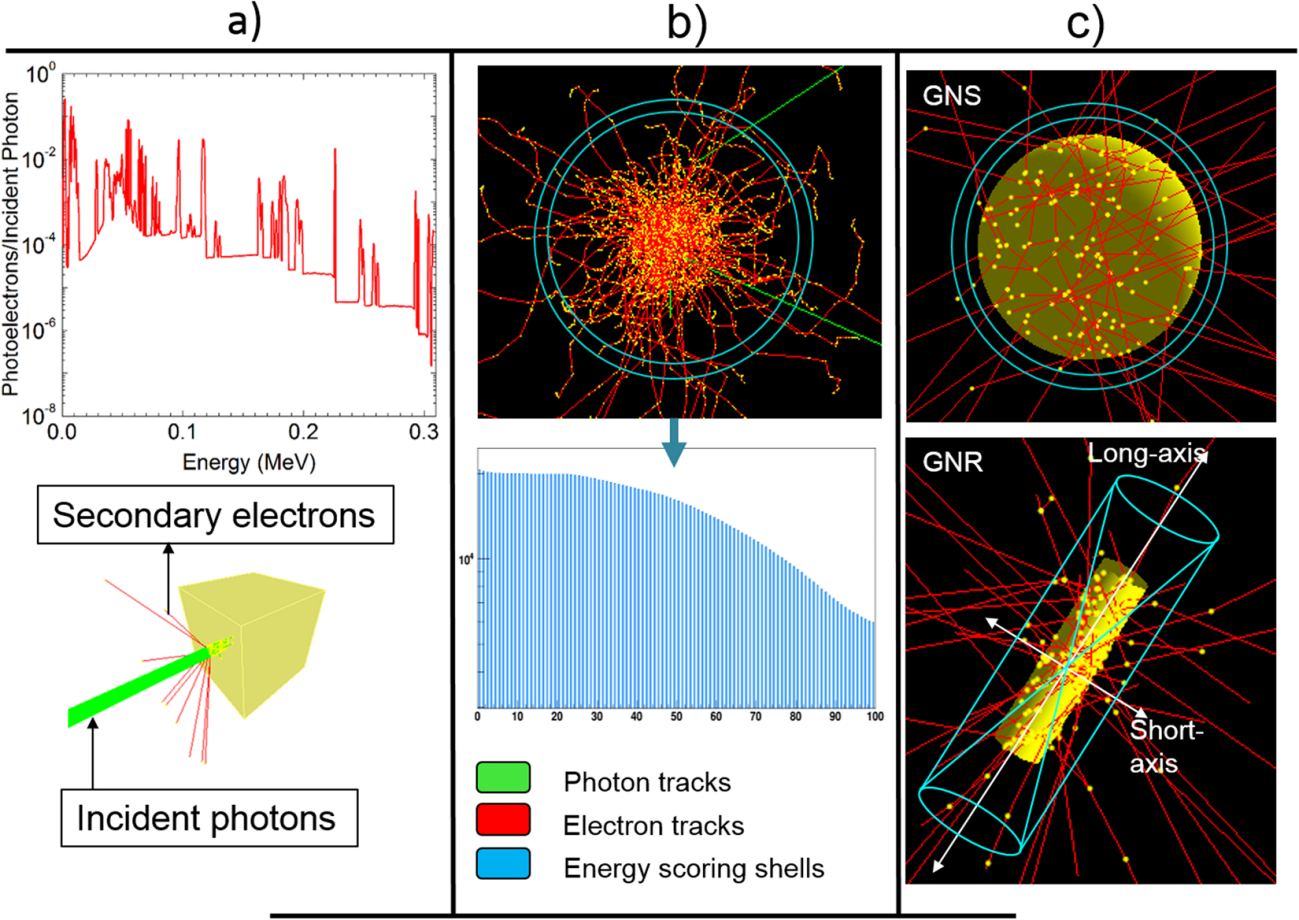
Multi-phase MC simulations. (**a**) Secondary electron spectra acquisition. (**b**) Derivation of the DPK. (**c**) Determination of the dose profiles for GNS and GNR.

We conducted the MC simulation in three phases, as illustrated in Fig. 7. In phase 1, we scored the secondary electrons’ kinetic energy due to the photon irradiation of a specific GNP geometry. A known polyenergetic incident photon spectrum (green) was used to derive the secondary electron spectrum (red). In phase 2, we generated the DPK by scoring the doses due to an isotropic point source, located at the center of a homogeneous water medium, emitting electrons with the energy spectrum derived from phase 1. In phase 3, we located the electron-emitting GNP sources at the center of the water medium and calculated the dose profiles around them. These dose profiles were then used to compare with the dose distributions calculated by applying the DPK rescaling methods.

#### Generation of the secondary electron spectra

A 3 mm × 3 mm × 3 mm gold cube situated in a vacuum was irradiated by two poly-energetic photon sources, Yb-169 and 6 MV, separately, as shown in Fig. 7(a). The size of a cubic phantom used in this task was large enough to ensure that virtually all photons available for photoelectric absorption (with gold) interacted within the phantom. A pencil beam of 1 mm diameter was used during the irradiation. The Yb-169 photon spectrum was obtained from a previous study^38^ and reproduced in Table 1. The 6 MV photon spectrum was obtained from another published study^39^. The kinetic energy of the secondary electrons produced inside the cubic geometry, due only to the first interactions between the incident photons and gold, was scored for the two incident photon sources separately in energy bins of 0.1 keV for the Yb-169 source and 1 keV for the 6 MV source. Two billion photon histories were used during the irradiation for each photon source.

**Table 1:** Yb-169 photon spectrum. Photon spectral lines with yields greater than 0.1% are shown.

Energy (MeV)	Photons per disintegration
0.04977	0.532
0.05074	0.940
0.05730	0.0993
0.05751	0.192
0.05790	0.00379
0.05903	0.0647
0.05921	0.0172
0.06312	0.442
0.09362	0.0261
0.10978	0.1747
0.11819	0.01869
0.13052	0.1131
0.17721	0.2216
0.19796	0.358
0.26108	0.01715
0.30774	0.1005
Total	3.32083

#### Derivation of the dose point kernel

The secondary electron spectrum was associated with an isotropic point source of electrons, which was placed at the center of a spherical water phantom with a 2 mm radius, as shown in Fig. 7(b). The current water phantom size was chosen to improve computational efficiency, while ensuring no statistically significant influence of the phantom size on the results. The total energy deposition due to each electron source was scored in 1 nm-thick concentric spherical shells with a maximum radius of 200 μm in the case of Yb-169 and 2 mm in the case of 6 MV. Specifically, the energy deposition of each interaction point in water was tabulated in a histogram as a function of radial distance. Two billion electrons were simulated separately for both spectra. To obtain the DPK, we divided the energy deposition in each shell (i.e., bin content of the histogram) by the mass of the appropriate spherical shell. At up to 100 μm from the origin, the statistical uncertainty was <0.6% for Yb-169 and <0.2% for 6 MV.

#### Estimation of dose profiles around GNPs

Point sources were replaced by GNSs of various radii, and the electron sources were randomly sampled within GNSs and set to emit electrons isotopically. For each simulation, the radial dose distribution was scored in water in 1 nm-thick spherical shells outside the GNS. For each geometry, two billion electrons were transported in gold and water media. The radial dose profile beyond the boundary of the GNS was obtained by dividing the energy deposition by the appropriate mass of the scoring shell. The statistical uncertainty during Yb-169 irradiation was <0.8% for a GNS with a radius of 5 nm and <1% for a GNS with a radius of 250 nm, up to 100 μm from the surface. In the case of 6 MV irradiation, the uncertainties were <0.3% for all GNS radii, up to 100 μm from the surface. This suggests that the produced secondary electrons from both photon sources have ranges long enough to deposit energy in all parts of a typical inter- or intra-mammalian (e.g., HeLa) cell geometry with a diameter of 20 μm^40^.

**Figure 8:**
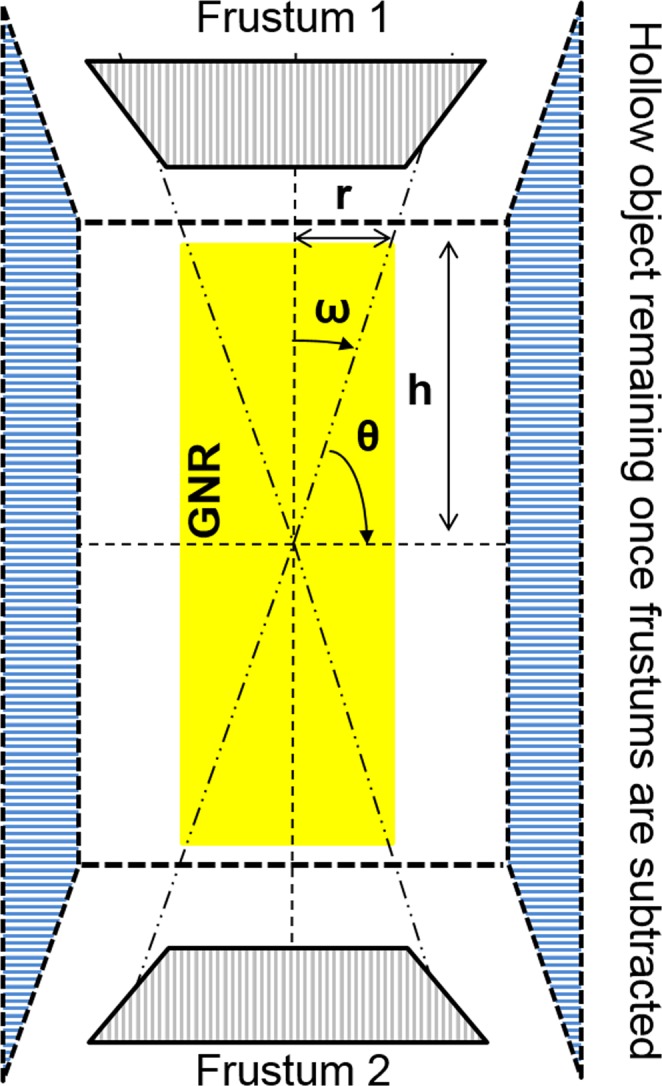
Scoring volumes for GNRs in planar and non-planar boundary systems in the z-x plane. Yellow indicates the GNR; gray indicates the two frustums; and blue indicates the hollow cylinder.

Finally, GNSs were replaced by GNRs of different aspect ratios. The whole GNR was assumed to be a combination of point sources of electrons and set to emit electrons isotropically. Unlike GNSs, which carry spherical symmetry, GNRs require an extraction of the dose distribution along each of the two axes with respect to the center of the coordinate system, namely the short-axis and long-axis (or main-axis) regions, as shown in Fig. 7(c). Let the half-length of the nanorod be ‘h’ and the radius be ‘r’, if the central axis of the rod is along the z-axis. We define the long-axis opening angle as $tan\,(\omega )=r/h$ and short-axis angle as $tan\,(\theta )=h/r$. If there is a phase space point (x, y, z) outside the GNR in such a way that $\sqrt{{x}^{2}+{y}^{2}}/|z| < \,\tan \,(\omega )$ and $|z| > h$, the point is considered to be in the long-axis region where electrons are crossing a planar boundary. On the other hand, if the point is restricted to the condition $|z|/\sqrt{{x}^{2}+{y}^{2}} < \,\tan \,(\theta )$ and $r < \sqrt{{x}^{2}+{y}^{2}}$, the point is considered to be in the short-axis region, and electrons are crossing a non-planar boundary. Once the proper location of a given point with respect to the long- or short-axis was identified, the energy deposition was tabulated for each point of the interaction during the simulation in the two frustums of a cone with a thickness of 1 nm for the long-axis and in a hollow cylinder  with a thickness of 1 nm derived from subtracting the two frustums in the corresponding cylinder for the short-axis (Fig. 8). To obtain the absorbed dose profile, the total energy deposition was divided by the mass of the appropriate scoring volume. For each considered aspect ratio, two billion electrons were simulated for both photon sources. The statistical uncertainties during Yb-169 irradiation were <1–3%, up to 100 μm, whereas during 6 MV irradiation, the uncertainties were <0.5–1%, for all considered aspect ratios.

### Analytical method

#### Geometry-based rescaling

**Figure 9:**
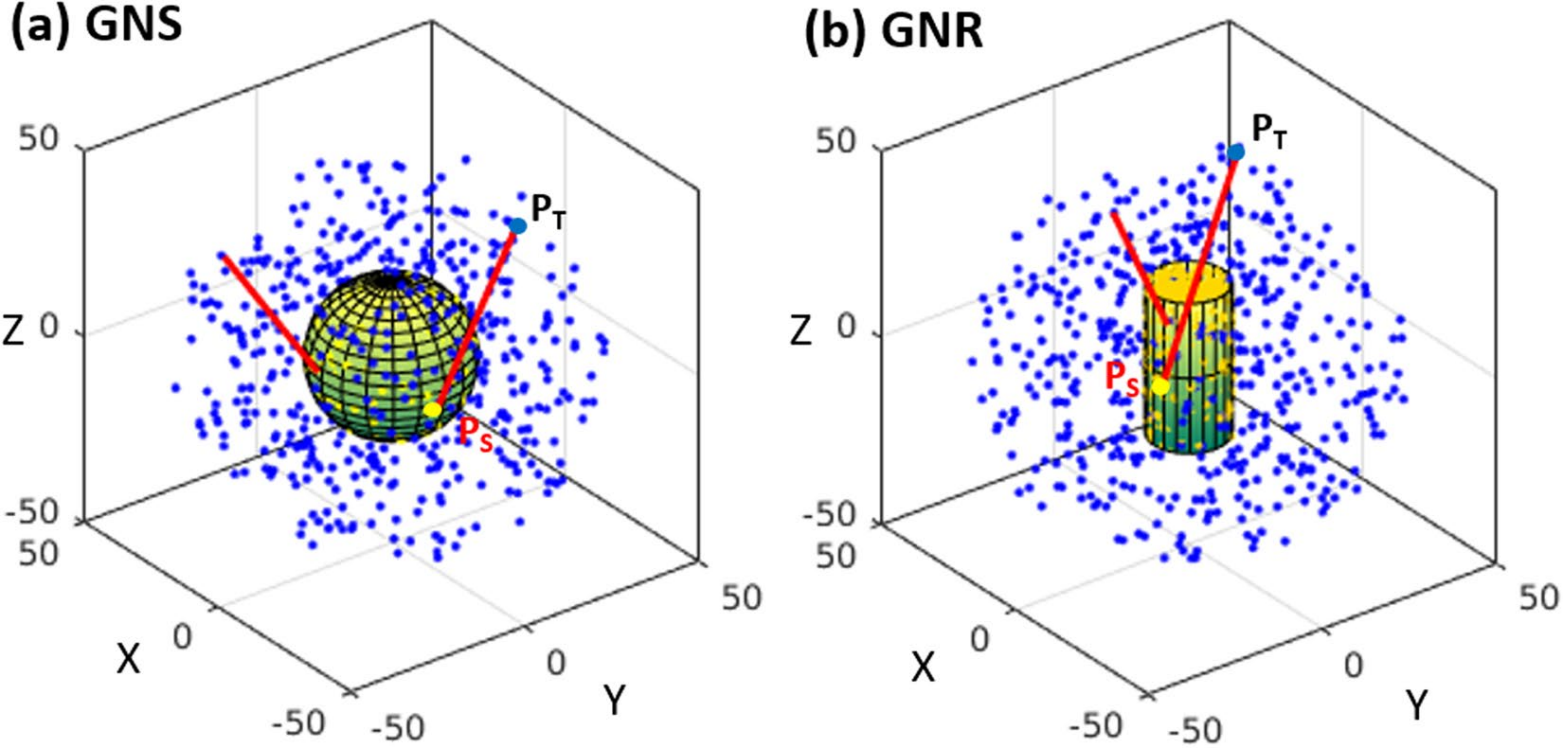
The EPA-based DPK rescaling method. (**a**) For GNSs, the SPs and TPs were randomly and uniformly sampled with respect to the origin of the coordinate system, and GPLs were derived. (**b**) For GNRs, the SPs and TPs were randomly and uniformly sampled with respect to the origin, and GPLs along the long- and short-axes were derived.

An electron-emitting GNP of finite size can be represented as a properly sampled distribution of point sources with source points (SPs). Similarly, the water medium outside the SPs can be modelled as properly sampled tally points (TPs). Since SPs would no longer be localized at the origin, the dose deposition outside the boundary of the GNP should be different from the results of the DPK. Once the distance between the SP and TP is known, the dose value at the TP due to the considered SP can be found by properly rescaling the original homogeneous DPK (in water). Analytical geometry can be used for each SP and TP combination to calculate the physical path length. The analytical simulation setups used to rescale the DPKs for GNSs and GNRs are illustrated in Fig. 9(a,b), respectively. First, either the GNS or GNR was constructed through uniform and randomly distributed SPs (yellow points) surrounded by water medium containing corresponding TPs (blue points). For GNR, the conditions for the long- or short-axis were examined after each TP was generated. Let P_S_(x_S_, y_S_, z_S_) be the coordinates of a point inside the GNP and P_T_(x_T_, y_T_, z_T_) be the coordinates of a point outside the GNP. For any SP-and-TP pair, the geometrical path length (GPL) is the distance between the points (the red tracks in Fig. 9(a,b)) and given by $\sqrt{{({x}_{S}-{x}_{T})}^{2}+{({y}_{S}-{y}_{T})}^{2}+{({z}_{S}-{z}_{T})}^{2}}$. The generation of SP and TP and the calculation of GPL were performed separately for all dimensional GNPs. Once the GPL was determined, it was used to integrate the dose contribution to the appropriate scoring shell using the derived homogeneous DPK. The sampling process was repeated for one billion phase space points (SP-and-TP pair) for all studied GNP geometries.

#### Material-based rescaling

In addition to geometrical rescaling, other adjustments may be applied to handle the heterogeneous situation. In principle, due to different energy absorption and scattering of the electrons as they traverse gold and water media, the dose distribution outside the GNP should be different from that due to the integration of the homogeneous DPK with only geometry-based rescaling. Therefore, a correction factor correlated with the distance traversed inside the GNP must be also considered. First, the distance traversed inside the GNP needs to be calculated for a given SP and TP. We constructed a system of equations using the Cartesian coordinate system for GNS or GNR. Let the distance traversed through the GNP be denoted by DTI (distance traversed inside). Considering a GNS with radius R_0_, along the path from SP to TP, there exists a unique point P_R_(x_R_, y_R_,z_R_) on the surface of the GNS where the distance from the center is equal to the radius and satisfies the equation ${{x}_{R}}^{2}+{{y}_{R}}^{2}+{{z}_{R}}^{2}={{R}_{0}}^{2}$. A parametric equation can be used to define any coordinate along P_S_ and P_T_ as ${x}_{\lambda }={{x}}_{s}\ast (1-\lambda )+{x}_{T}\ast \lambda$, where λ (0 ≤ λ ≤ 1) is a parameter to be found. The same type of equation can be defined to y, z coordinates and, substituting for the previous equation, a system of quadratic equations can be found to solve for λ. Substituting λ back to the parametric equation will give the appropriate coordinates of P_R_, and the DTI can be found as $\sqrt{{({x}_{S}-{x}_{R})}^{2}+{({y}_{S}-{y}_{R})}^{2}+{({z}_{S}-{z}_{R})}^{2}}$. In the case of GNRs, for the short-axis region, the same procedure used for GNSs can be followed, while for the long-axis region the parametric equation is simplified to ${z}_{\lambda }={z}_{R}\ast (1-\lambda )+{z}_{T}\ast \lambda =h$. This will be directly solved for λ, and substituting back into the parametric equations gives the coordinates of P_R_. The electron inside the GNP undergoes much more scattering, and more energy is transferred to the GNP than to homogeneous water. Therefore, it can be assumed that traversing a certain distance inside the GNP is equivalent to a much longer linear distance in water. Once the DTI is properly identified for a given geometry, the modified path length (MPL) is defined instead of GPL using the equation $MPL=GPL+(\alpha -1)\ast DTI$. Here, α (α ≥ 1) is a dimensionless rescaling factor between the GNP and surrounding medium. Once the modified path length is calculated using the appropriate α for a heterogeneous medium, the absorbed dose at a TP for a particular source point of a GNP can be estimated by integrating the unperturbed DPK in water.

#### Unity rescaling factor (no rescaling)

This is independent of the material (gold) and the interactions of the secondary electrons, and is to be taken as α = 1. In this scenario, the MPL becomes the GPL, and only the geometry-based rescaling is applied during the analytical simulation.

#### TSP rescaling factor

We defined TSP rescaling factor as the energy spectrum-weighted ratio of TSP in gold to water for the derived secondary electron spectra of either Yb-169 or 6 MV. This factor incorporates both the medium and the interactions (causing energy loss) of the secondary electrons:

1 $$TSP=\frac{\sum {E}_{i}\cdot TS{P}_{Au}^{{E}_{i}}}{\sum {E}_{i}\cdot TS{P}_{Water}^{{E}_{i}}}.$$

Here, $TS{P}_{Au}^{{E}_{i}}$ and $TS{P}_{Water}^{{E}_{i}}$ are the TSP in gold or water for a corresponding energy bin of *E*_*i*_ from the obtained secondary electron spectra of either Yb-169 or 6 MV.

#### LRR rescaling factor

The LRR rescaling factor was computed as the ratio of energy spectrum-weighted CSDA ranges in water to gold using the energy spectrum of the obtained secondary electrons from gold for each photon source:

2 $$LRR=\,\frac{\sum {E}_{i}\cdot CSD{A}_{Water}^{{E}_{i}}}{\sum {E}_{i}\cdot CSD{A}_{Au}^{{E}_{i}}}.$$

Here, $CSD{A}_{Au}^{{E}_{i}}$ and $CSD{A}_{Water}^{{E}_{i}}$ are the CSDA range in gold or water for an energy bin of ${E}_{i}\,$from the obtained secondary electron spectra of either Yb-169 or 6 MV.

#### PDR rescaling factor

Another alternative is to use the ratio of the physical densities of the two materials. In the current study of water and gold, it is 19.3. This is a rescaling factor that depends on only the material properties. Different rescaling factors for both incident photon sources are summarized in Table 2.

**Table 2:** Summary of the different rescaling factors obtained for two different incident photon sources.

	Unity	TSP	LRR	PDR
Yb-169	1.0	8.2	9.6	19.3
6 MV	1.0	14.0	13.0	19.3

#### Comparison of rescaling factors

To quantify the matching of the G4-simulated dose profiles with different DPK rescaling methods, we calculated the mean absolute percentage error (MAPE), which is defined as:

3 $$MAPE=\frac{100}{N}\sum _{i=1}^{N}|\frac{{G}_{i}(r)-{S}_{i}(r)}{{G}_{i}(r)}|.$$

Here, G_i_(r) is the corresponding absorbed dose at distance r derived from the G4 simulation, and S_i_(r) is the absorbed dose at the same distance obtained by a corresponding DPK rescaling factors for N number of radial points from the surface of a particular geometry.

### Dosimetry calculations with clustered GNPs within a cell

**Figure 10:**
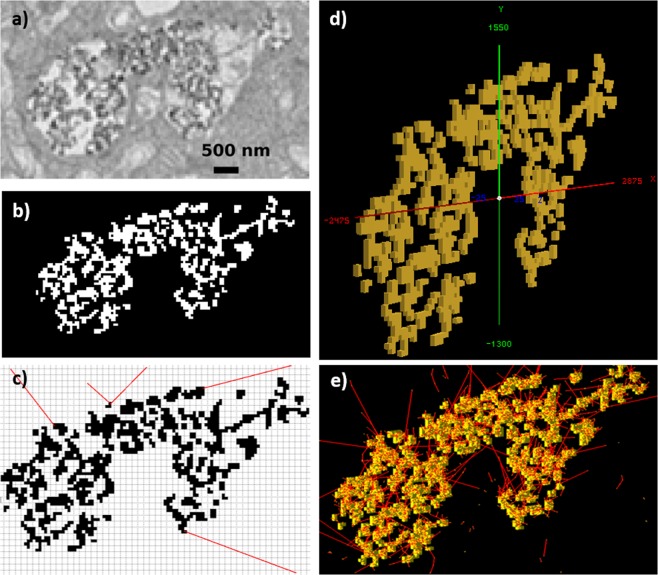
Dosimetry calculations with internalized GNP clusters obtained using a TEM image. (**a**) Original TEM image with GNPs accumulated inside the cellular endosome. (**b**) Extraction of the GNP pixels via applying a threshold pixel value. (**c**). DPK rescaling method for realistic 2D GNP clusters on a pixelated grid via ray tracing. (**d**) 3D modelling of the GNP clusters as an input geometry into G4 toolkit. (**e**) Full-fledged G4 MC simulation for scoring the energy deposition in water medium due to secondary electron emission (red) from GNPs when irradiated with photons.

To investigate the applicability of the DPK rescaling method in a realistic scenario of clustered GNPs, we pre-processed a TEM image of a head & neck cancer cell containing internalized GNPs, to create a test case with the input geometry, as shown in Fig. 10(a).

The TEM image was 6400 nm × 3650 nm and consisted of 9344 pixels of either gold or water; the individual pixel size was 50 nm. Once the TEM image had been imported, a threshold based on the pixel RGB values (<0.4) was applied to separate GNP pixels from the water. In this study, 1304 gold pixels and 8040 water pixels were separated for subsequent analysis as shown in Fig. 10(b). A full-fledged G4 MC simulation was designed to compare the results obtained from the DPK rescaling method.

#### Full-fledged G4 MC simulation

Once the GNP pixels and water pixels were properly distinguished, the image was voxelized to a G4-compatible geometry input file. Since MC simulations cannot be run in two-dimensional geometries, a height of 50 nm was selected for the z-axis, and the final dimensions of a single voxel were 50 nm × 50 nm × 50 nm (Fig. 10(d)). The geometry file was input into a custom G4 simulation, and Yb-169 & 6 MV photon spectra were induced in the realistic GNP geometry. The energy deposition due to emitted secondary electrons was scored in water voxels (Fig. 10(e)). Sixty billion photon histories were simulated. To obtain the absorbed dose distribution, the energy deposition was divided by the mass of the corresponding water voxel.

#### DPK-based analytic calculations

Each GNP pixel was assumed to be an isotropic electron point source (SP). All other pixels were assumed to be water pixels (TPs). Electron tracks were generated from each SP to the rest of the TPs, as shown in Fig. 10(c), and the dose deposition was calculated using the original homogeneous DPK based on the distance between two points. The Bresenham ray tracing algorithm^41^ was utilized in order to find any GNP pixels intercepted between the SP and TP. This gives the DTI in the pixelated GNP cluster, which can be used with any rescaling factor to correct for energy loss during electron transport through GNP pixels. After proper rescaling and calculating the dose for a particular water pixel, single kernel superposition was used to calculate the total dose deposition in each water pixel from every other GNP pixel.

## Supplementary information

Supplementary Information
