# Supplementary material for: Evaluation of dose point kernel rescaling methods for nanoscale dose estimation around gold nanoparticles using Geant4 Monte Carlo simulations
Source: Sci Rep. 2019 Mar 5;9:3583. doi: 10.1038/s41598-019-40166-9 (PMC6401138; doi:10.1038/s41598-019-40166-9)
Supplement: Supplementary file 1 — Supplementary Information [file 41598_2019_40166_MOESM1_ESM.pdf]

Supplementary Information

Evaluation of dose point kernel rescaling methods for nanoscale dose estimation around gold nanoparticles using Geant4 Monte Carlo simulations

Sandun Jayarathna, Nivedh Manohar, Md Foiez Ahmed, Sunil Krishnan, Sang Hyun Cho

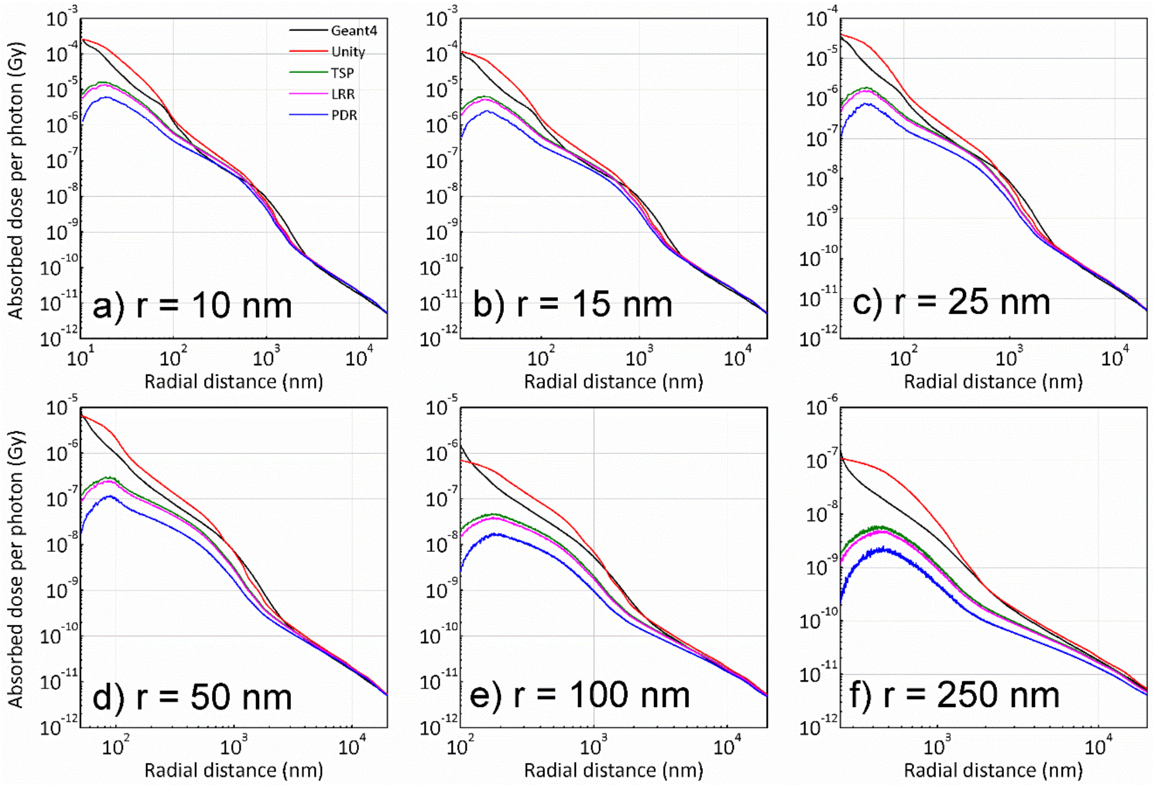

Supplementary Figure S1. Comparison of the absorbed dose distribution around GNSs as assessed by MC and DPK/rescaling methods for Yb-169 irradiation for the rest of the geometries.

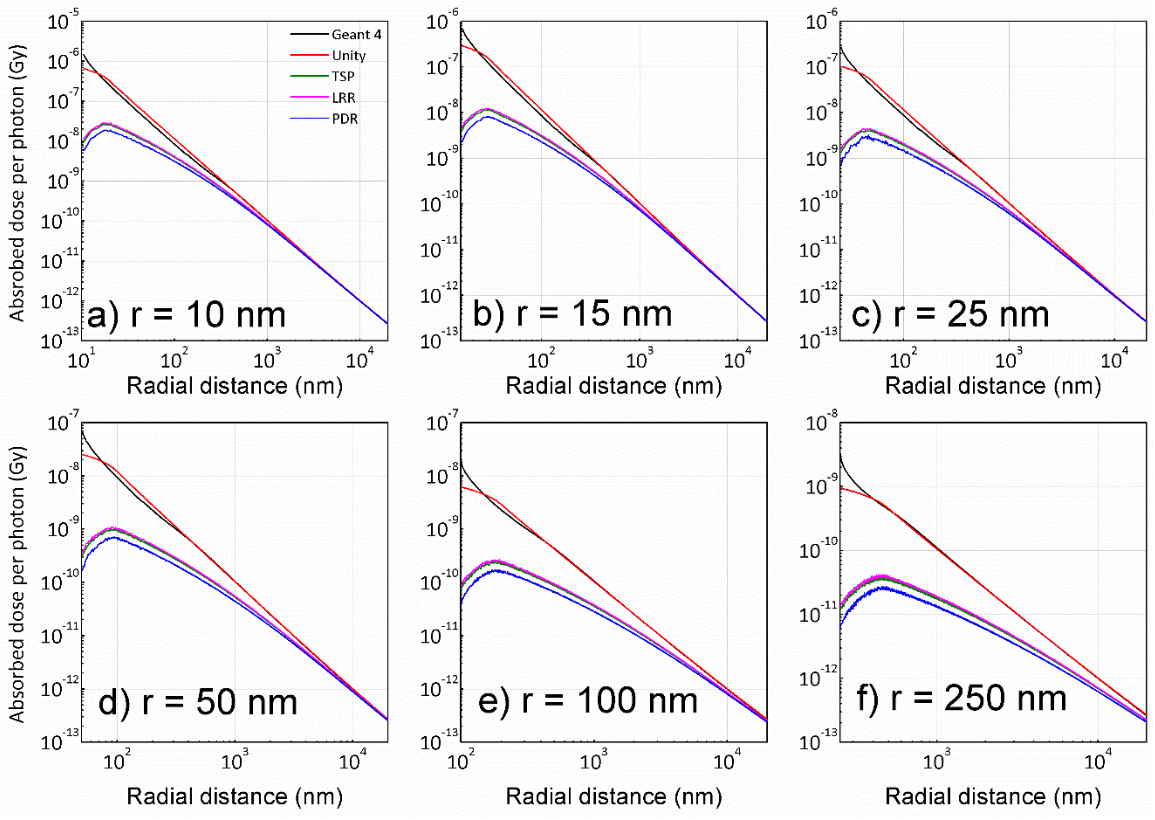

Supplementary Figure S2. Comparison of the absorbed dose distribution around GNSs as assessed by MC and DPK/rescaling methods for 6 MV irradiation for the rest of the geometries.

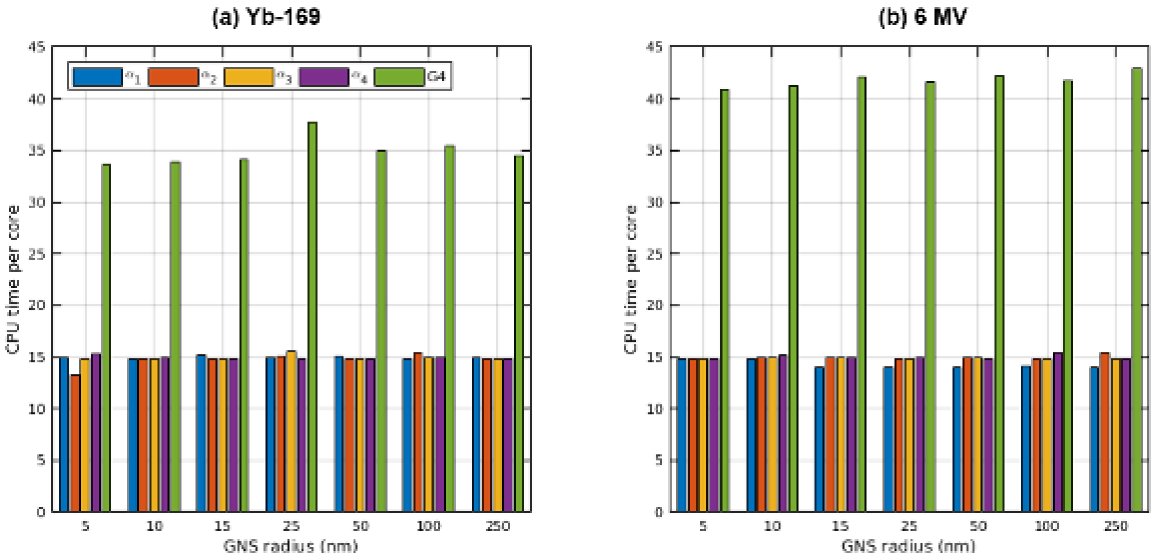

Supplementary Figure S3. Processing times for GNS geometries for different DPK/rescaling methods and G4 simulations in (a) Yb-169 irradiation and (b) 6-MV irradiation.

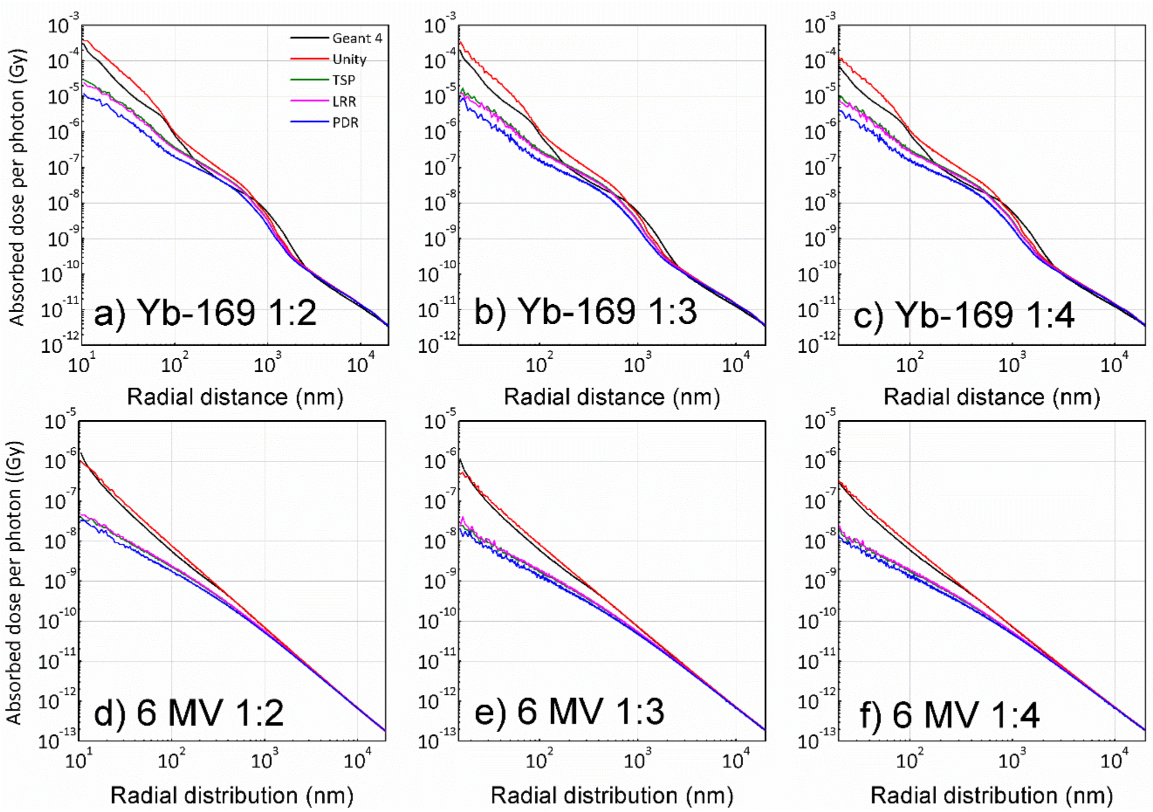

Supplementary Figure S4. Comparison of the absorbed dose distribution around the planar boundaries of GNRs by MC and DPK/rescaling methods for Yb-169 irradiation (a,b,c) and 6-MV irradiation (d,e,f) for the rest of the aspect ratios.
